# Health Status and Preventive Health Services Among Reproductive-Aged Women in Treatment for Opioid Use Disorder

**DOI:** 10.1089/whr.2022.0057

**Published:** 2022-12-15

**Authors:** Vanessa L. Short, Dennis J. Hand, Lauren Pyfer, Hanna Steiger, Meghan Gannon, Gregory Jaffe, Diane J. Abatemarco

**Affiliations:** ^1^ College of Nursing, Thomas Jefferson University, Philadelphia, Pennsylvania, USA.; ^2^ Sidney Kimmel Medical College, Thomas Jefferson University, Philadelphia, Pennsylvania, USA.

**Keywords:** opioid use disorder, survey, women, preventive health, health status, chronic disease

## Abstract

**Objective:**

To assess the utilization of preventive health services and the prevalence of chronic health conditions among a cohort of women in treatment for opioid use disorder (OUD).

**Methods:**

Ninety-seven women who were receiving treatment for OUD from a single urban treatment program completed a self-administered anonymous online questionnaire that asked about demographics, health, receipt of preventive health services, and utilization of health care. Descriptive statistics were used to describe data.

**Results:**

More than one-third of respondents reported that their health was fair or poor, whereas one-quarter were very concerned with their health. Most participants (59%) reported at least one chronic health condition; nearly 1 in 5 reported two or more conditions. Less than half of respondents had received a routine medical examination in the past year. Vaccine uptake was low; 56% received the coronavirus disease 2019 vaccine and 36% received the annual influenza vaccine.

**Conclusions:**

Women in treatment for OUD could benefit from enhanced health care to address the high rates of chronic diseases and risk factors and underutilization of recommended preventive health services. Interventions and models of care that aim to enhance utilization of such services, and ultimately improve the health of this vulnerable population, may be worth exploring.

## Introduction

The opioid crisis is a major public health issue in the United States. More than 130 people a day die from opioid-related drug overdoses in the United States,^1^ and opioid use disorder (OUD) is associated with substantial and increasing hospitalizations and health care costs across the country.^2,3^

Notably, OUD has increased in prevalence among reproductive-aged women, having quadrupled since the late 1990s among pregnant women in the United States.^4^

Reproductive-aged women with OUD often need a range of medical, behavioral health, and social services.^5^ Chronic diseases (*e.g*., chronic obstructive pulmonary disease [COPD]), infectious diseases (*e.g*., Hepatitis C, HIV), and risk factors associated with suboptimal health (*e.g*., tobacco use) tend to be prevalent in this population,^6–9^ whereas use of preventive health measures (*e.g*., contraception use) is low, suggesting that they have unmet health care needs. Moreover, the gold standard of treatment for OUD includes comprehensive behavioral and medical services combined with opioid agonist medications. Yet, opioid agonists can adversely impact health as they are known to increase insulin resistance and are associated with metabolic disease.^10^ Finally, factors that strongly influence health behaviors, decisions and outcomes, and access to quality health care, including poverty, low educational attainment, stigmatization, stress, and history of trauma, are common in the population.^11–14^

It has been previously reported that individuals with OUD often have limited access to and engagement with primary care,^15,16^ which is an important aspect of one's own health as it allows for monitoring of conditions, and provides opportunities for preventing and diagnosing illness and injury, guidance to reduce risk factors and optimize health outcomes, and coordination of testing and specialist care if needed. Factors such as socioeconomic and psychosocial determinants of health as well as stigma and discrimination are common among women with OUD and may further limit the utilization of primary care and important preventive services, such as vaccinations and health screenings.

The specific preventive health needs of women with OUD, however, have not been adequately described, limiting the ability of interventionists and policy makers to make informed decisions about strategies to improve the health of this patient population. Therefore, the main objectives of this exploratory study were to describe the receipt of preventive health services among a cohort of women in treatment for OUD and to assess the prevalence of chronic health conditions in this population. It is anticipated that results from this survey-based study will inform the design of innovative interventions and models of care for women with OUD that address their unmet health needs and gaps in recommended preventive health care and services.

## Methods

Data for this cross-sectional study were collected between September and November 2021 from a sample of women receiving treatment for OUD from a single urban opioid treatment program that serves pregnant and/or parenting women exclusively. The opioid treatment program is affiliated with a university health system and was part of the university's obstetrics and gynecology department at the time of the survey. The opioid treatment program is licensed to provide both methadone and buprenorphine for OUD. Other services, including group and individual therapy, psychiatric care, case management, and advocacy for social needs (*e.g*., housing and vocational), are provided at the opioid treatment program. Given the program's specific focus on pregnant and/or parenting women, participants received coordination of prenatal care through the program's affiliated obstetrics and gynecology department. At the time of the survey, the opioid treatment program did not provide primary care services, and the only preventive health screenings conducted were annual routine bloodwork as required for opioid treatment programs. These annual tests included a complete blood count, comprehensive metabolic panel, hepatitis B titers, hepatitis C antibody and polymerase chain reaction, and tests for syphilis. Patients with abnormal results received referrals to specialty providers. Information about whether these referrals resulted in future care was not collected by the program. The opioid treatment program conducted a 2-day free influenza vaccine drive during the survey period run by the affiliated university's college of nursing. The only study criteria for exclusion were age <18 years and not being able to read and write English. Participation in the study was voluntary and the appropriate Institutional Review Boards approved all study procedures. The data that support the findings of this study are available from the corresponding author upon request.

To recruit participants, a study flyer was posted in the treatment program's waiting area. The flyer contained contact information for research personnel and a quick response (QR) code. Individuals had the option to scan the QR code with a smart phone to access the Qualtrics questionnaire or to contact research personnel for the Qualtrics link. Participants also had the option to complete the survey on an iPad provided by research personnel. Subjects provided consent to participate in the study by typing their name on the first page of the online questionnaire. Each participant had the option of receiving $20.00 USD on a Clincard (a reloadable debit card) after completing the survey. Those who wanted to receive the remuneration provided their name and phone number after completing the questionnaire and research personnel coordinated distribution of the Clincard.

The questionnaire contained a total of 80 items and used, to the extent possible, items from the Behavioral Risk Factor Surveillance System, a system of surveys that collect state-level data about U.S. residents regarding their health-related risk behaviors, chronic health conditions, and use of preventive services.^17^ Questionnaire items asked participants to report demographic and health characteristics, including age, race, ethnicity, employment status, relationship status, highest level of education, pregnancy status, and overall health status. Participants were also asked if they had ever been told by a doctor or health care provider that they had high blood pressure, obesity, prediabetes, diabetes, COPD, or Hepatitis C (yes/no). Based on their responses, participants were identified as having 0, 1, or ≥2 conditions.

Participants were also asked about receipt of preventive health services (yes/no) including cervical cancer screening (*i.e*., Pap test) within the past 3 years, routine medical examination (checkup) within the past year, influenza vaccine within the past year, and coronavirus disease 2019 (COVID-19) vaccine. Utilization of health care was also assessed, including place most often visited when sick (emergency room, urgent care clinic, personal doctor, community, or other clinic). Those who responded that they had not received a routine examination in the past year or had not received influenza vaccine in the past year were asked about the last time they had a routine medical examination or influenza vaccine (1–2 years, 2–5 years, 5 or more years, and never). Those who responded that they had not received the COVID-19 vaccine were asked about intention to get the vaccine with the follow-up question: “Will you ever get the COVID-19 vaccine?” (yes, no, and not sure).

Data were downloaded from Qualtrics and analyzed using SAS version 9.4 (SAS Institute, Cary, NC). The final analytic data set did not include participants name or contact information. Descriptive statistics, including frequency counts and percentages for categorical data and medians and ranges for continuous data, were used to describe data.

## Results

Ninety-seven individuals completed the questionnaire, which represented ∼54% of the patient population at the opioid treatment program at the time of survey distribution. Six (6%) individuals reported being pregnant at time of questionnaire completion. Table 1 shows the demographic characteristics of study participants. Median age at time of survey completion was 34 years (range 26–63). Most respondents were white (66%), non-Hispanic (90%), not employed (81%), not married (84%), and had no more than a high school education (67%). The majority of participants (83%) smoked cigarettes and/or vaped.

**Table 1.** Self-Reported Demographic Characteristics of Study Participants

	***n* (%)**
Age in years, median (range)	34 (26–63)
Age in years	
26–29	11 (13)
30–39	60 (68)
40–49	16 (18)
≥50	2 (2)
Race	
Black	23 (24)
White	64 (66)
Other^a^	10 (10)
Ethnicity	
Hispanic	10 (10)
Non-Hispanic	86 (90)
Highest level of education	
Less than high school	26 (27)
High school graduate	39 (40)
Some college or college	32 (33)
Marital status	
Married	15 (16)
Not married	81 (84)
Employment status	
Employed full time	12 (12)
Employed part time	7 (7)
Not employed	78 (81)
Number of children at home	
0	10 (10)
1	37 (38)
2	21 (22)
3 or more	29 (30)
Current smoker	
Yes, cigarettes	71 (73)
Yes, vape	26 (27)
None	17 (17)

^a^ Includes participants who reported more than 1 race.

Table 2 shows the prevalence of self-reported health conditions and utilization of preventive health services. More than one-third of respondents reported that their health was fair or poor, whereas one-quarter were very concerned with their health. Most participants (59%) reported at least one chronic health condition; nearly 1 in 5 reported 2 or more conditions. The most commonly self-reported health conditions included Hepatitis C (32%), high blood pressure (21%), and obesity (14%). When asked about the place most often visited for a sick visit, 36% reported that they went to the hospital emergency room, 35% reported that they went to an urgent health clinic, and 25% reported that they saw their own personal doctor.

**Table 2.** Self-Reported Health and Utilization of Health Services

	***n* (%)**
Overall health	
Excellent or very good	24 (24)
Good	36 (38)
Fair or poor	36 (38)
Concern for own health	
Very concerned	23 (23)
Somewhat concerned	57 (59)
Not at all concerned	17 (17)
Personal primary care provider	
Yes	59 (61)
No	37 (39)
Place most frequently visited when sick	
Emergency room	35 (36)
Urgent health clinic	34 (35)
Personal doctor	24 (25)
Community or other health clinic	4 (3)
Health conditions	
Hepatitis C	31 (32)
High blood pressure	20 (21)
Obesity	14 (14)
COPD	9 (9)
Diabetes	6 (6)
Prediabetes	6 (6)
Number of health conditions^a^	
0	40 (41)
1	39 (40)
2 or more	18 (19)
Time since last regular checkup^b^	
1–2 years	24 (47)
2–5 years	17 (33)
>5 years	7 (14)
Never	3 (6)
Time since last influenza vaccine^c^	
1–2 years	29 (44)
2–5 years	12 (18)
>5 years	11 (17)
Never	14 (21)

^a^ Conditions include Hepatitis C, high blood pressure, obesity, COPD, diabetes, and prediabetes.

^b^ Among those who reported no annual checkup in the past year.

^c^ Among those who reported no influenza vaccine in the past year.

COPD, chronic obstructive pulmonary disease.

**FIG. 1.**
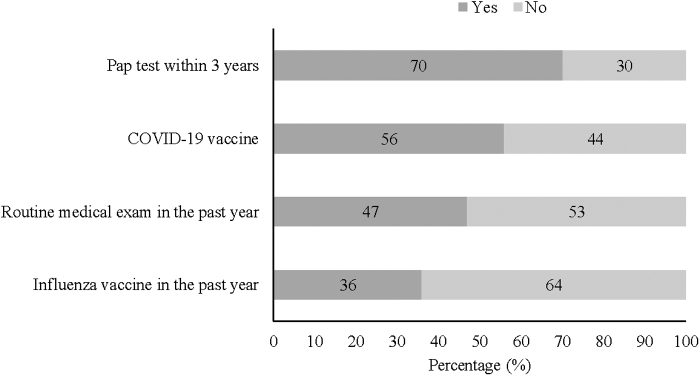
Self-reported receipt of preventive health services.

Among the preventive health services asked about, receipt of on-time cervical cancer screening was the most frequently reported, followed by receipt of the COVID-19 vaccine (Fig. 1). Less than half of respondents had received a routine medical examination in the past year. Among those who had not received a routine annual examination, more than half had not had one in two or more years (Table 2). One-third of respondents reported that they had received the influenza vaccine in the past year. Among those who had not received an annual influenza vaccine, 21% had never received the vaccine.

**FIG. 2.**
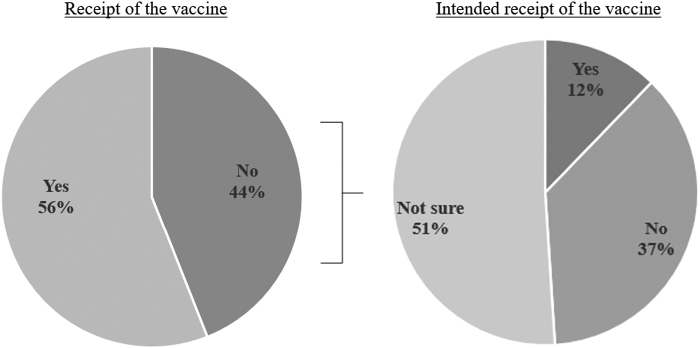
Receipt and intended receipt of the COVID-19 vaccine. COVID-19, coronavirus disease 2019.

Slightly more than half of respondents reported that they had received the COVID-19 vaccine (Fig. 2). Of those who had not been vaccinated at time of survey completion, 12% reported that they planned to get vaccinated, 37% reported that they did not plan to be vaccinated, and 51% were unsure if they would ever get the vaccine. Among the six respondents who reported being pregnant at time of survey completion, five (83%) received the vaccine.

## Discussion

Findings from this study suggest that there is a need for enhancing and for proactively providing preventive health services for women in treatment for OUD to address patient concerns, the high prevalence of chronic health conditions, and the underutilization of recommended screenings and vaccines. The rates of health conditions, including high blood pressure, COPD, and diabetes, and risk behaviors, including tobacco use, were higher than rates recently reported in the United States adult female population.^18^ This is not unexpected given the socioeconomic demographic profile of our study sample and the reported association between health and sociodemographics, such as educational attainment and employment status.^19^ Other factors that strongly influence health behaviors, decisions and outcomes, including poverty, stigmatization, stress, and trauma, are also common in women with OUD.^11–14,20^ Moreover, suboptimal health knowledge around specific areas (*e.g*., breastfeeding and contraception^21,22^), which can impact behaviors and outcomes, has also been previously reported in this patient population. In addition, few women in this study rated their own health as excellent or very good. This is concerning given that self-assessed health status is a strong measure of overall health status and is correlated with health service use, functional status, and mortality.^23^

This study also suggests that adult women with OUD do not frequently receive or utilize preventive health services beyond those recommended for obstetrical/gynecological health. For example, 70% of respondents reported receiving a cervical cancer screening within the recommended time frame, which is similar to rates among female buprenorphine patients^24^ and to the general female population in the United States.^25^ Less than half of participants, however, received a recent annual checkup, compared with 82% of U.S. female adults in 2020.^26^ All but one of the pregnant respondents in this study reported receiving the influenza or COVID-19 vaccine within the past year, whereas only one-third of nonpregnant respondents reported receiving the influenza vaccine and approximately one-half reported receiving the COVID-19 vaccine. It could be that women have more opportunities during pregnancy to receive health information given the frequency of prenatal care visits, and not being pregnant results in not having an established health care provider beyond the Ob/Gyn office. Evidence for this is provided by this study's data showing that nearly three quarters of participants reported that they go to the emergency room or urgent care when sick, whereas less than one-quarter visit their own personal doctor.

Another important finding from this study was that only slightly more than half of respondents reported COVID-19 vaccine receipt at a time when multiple vaccines were widely available for adults in the United States. According to the Centers for Disease Control and Prevention, nearly 65% of adults had received at least one dose of a COVID-19 vaccine during the time our survey was administered.^27^ Our findings highlight the need to identify reasons for low vaccine uptake in our patient population, as individuals with OUD are at significantly increased risk for COVID-19 and its adverse outcomes.^28^ Moreover, since most participants in this study were of reproductive age and likely had the potential to become pregnant, vaccine uptake may be especially important given that pregnant persons are at risk for severe COVID-19,^29,30^ COVID-19 is associated with adverse birth outcomes,^30^ and maternal vaccination protects the developing fetus and infant from infection during the first months of life.

The high rates of chronic health conditions and low uptake of preventive health services suggests a need to modify current approaches to health care delivery for women with OUD. Although integrating OUD treatment into primary care clinics has been shown to be feasible and positively impact both OUD treatment and health outcomes,^24,31,32^ that strategy alone will not meet the needs of all. The present results suggest that many women in treatment for OUD do not have already established relationships with health care providers and do not utilize primary care regularly. Moreover, it is common for persons with OUD to experience significant social barriers to accessing primary care (*e.g*., poverty and lack of childcare).

Alternately, integrating preventive health services into opioid treatment programs, such as offering health education, physicals, screenings, and/or vaccines on-site, may be a more effective option for several reasons. First, persons with OUD attend the opioid treatment program frequently so offering health services on-site would increase access and convenience. At the program where this study was conducted, the vast majority of participants came to the program daily for methadone dispensing, counseling, and/or other treatment activities. Seeking treatment for OUD could serve as an entry point to receive other health services. Second, treatment program clinicians (*e.g*., nurses) are already trusted by treatment program patients and, therefore, could be used to deliver accurate evidence-based health information and services. Having an established provider would allow for personalized care and enhance continuity of care, two aspects of clinical care that are valued by women in treatment for OUD.^33^

Although there is limited literature on the successful implementation of comprehensive preventive health services within OUD treatment programs, the integration of preventative services for infectious diseases, such as HIV and viral hepatitis, has been reported in treatment settings in the United States,^34^ suggesting that such integration would be feasible. Patient acceptability of colocating other types of health care, such as well child care and midwifery, within OUD treatment programs has been reported.^35,36^ Implementation research examining the feasibility and effectiveness of integrating preventive health services and care within substance use treatment programs is needed.

In addition to the convenience sample and the small number of pregnant respondents, other potential limitations to the study are noted. All participants were recruited from a single OUD treatment program located in an urban setting. Future studies with a larger and/or more diverse sample could allow for an analysis of the impact of individual-level characteristics on outcomes. It is possible that social desirability bias may have been present, although this was likely minimal given the questionnaires were self-administered and anonymous. Recall bias may have occurred given participants were asked to report on past events and behaviors. Data were collected during the COVID-19 pandemic, and it is possible that the pandemic influenced respondent reported behaviors, particularly those related to health care utilization. Use of existing questionnaire items to collect not previously reported information about a range of topics was a strength of the study.

## Conclusion

In summary, this exploratory study suggests that women in treatment for OUD have concerns for their own health and could benefit from enhanced preventive health care to address the high rates of chronic health conditions and risk behaviors and low rates of optimal health behaviors also present in this population. Examining patient-, provider-, and system-level barriers and facilitators that influence preventive health practices, including the uptake of vaccines and utilization of recommendation health screenings, could be an important next step to inform interventions, programs, and policies that address the needs of women with OUD. Finally, future studies may be warranted to test innovative models of care and preventive health programs that aim to enhance the health of this vulnerable population.

## Abbreviations Used

COPD: chronic obstructive pulmonary disease
COVID-19: coronavirus disease 2019
OUD: opioid use disorder
QR: quick response
